# Early Post-liver Transplantation Fever in a Child

**Published:** 2012-05-01

**Authors:** B. Geramizadeh

**Affiliations:** *Department of Pathology, Transplant Research Center, Shiraz University of Medical Sciences, Shiraz, Iran*

A2.5-year-old girl underwent orthotopic liver transplantation (OLT) for cirrhosis secondary to progressive familial intrahepatic cholestasis. She was well until five days post-transplantation when she developed fever (up to 39 °C). At that time, she was receiving tacrolimus, cellcept and prednisolone. Her laboratory findings revealed a serum ALT of 70 IU/L, AST of 75 IU/L, Alk-P of 560 IU/L, and a total bilirubin of 2 mg/dL with a direct bilirubin of 0.7 mg/dL. Epstein-Barr virus capsid antigen and cytomegalovirus IgM were negative. Blood culture for fungus and bacteria were also negative. Photomicrograph of the biopsy is shown in Figure 1.

**Figure 1:**
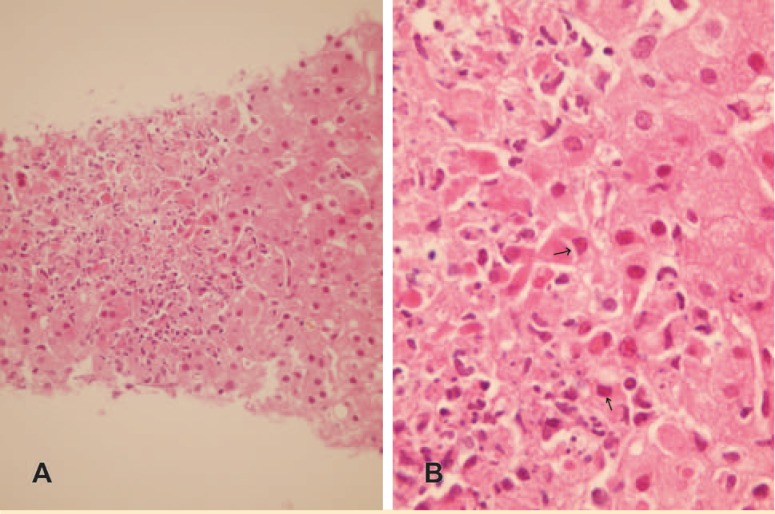
Sections from liver allograft 25 days after liver transplantation. A) Low power (H&E ×250) B) High power (×400)

## WHAT IS YOUR DIAGNOSIS?

Diagnosis: **Herpes Simplex Viral Hepatitis**

Herpes simplex (HSV) viral hepatitis is very rare in immunocompetent individuals [1]. Most of the cases have been reported in immunocompromised patients such as organ transplant recipients [2]. HSV hepatitis in transplanted liver is also rare and usually occurs as early as five days post-liver transplantation (median: 18 days) [3]. HSV infection is mostly due to the reactivation of a latent virus [4]. It seems that HSV hepatitis, as an early event, is most likely transmitted by the transplanted organ [5].

Though difficult, early diagnosis of HSV infection is very important. It can be diagnosed by PCR, isolation of the virus, and histopathology [4]. Histopathology of HSV hepatitis is characteristic—*i.e.*, well-defined foci of necrosis (Fig 1), at the edge of which hepatocytes exhibit nuclei with amphophilic viral inclusions. (Fig 1b, arrow) The inclusion bodies can be confirmed by immunohistochemical staining. The treatment of choice for HSV hepatitis is acyclovir [5]. Due to early diagnosis and prompt treatment, our patient survived and is doing well.
